# Can daily intake of aspirin and/or statins influence the behavior of non-muscle invasive bladder cancer? A retrospective study on a cohort of patients undergoing transurethral bladder resection

**DOI:** 10.1186/s12885-015-1152-x

**Published:** 2015-03-13

**Authors:** Antonio Luigi Pastore, Giovanni Palleschi, Andrea Fuschi, Luigi Silvestri, Yazan Al Salhi, Elisabetta Costantini, Alessandro Zucchi, Vincenzo Petrozza, Cosimo de Nunzio, Antonio Carbone

**Affiliations:** 1 Department of Medico-Surgical Sciences and Biotechnologies, Sapienza University of Rome, Faculty of Pharmacy and Medicine, Urology Unit ICOT, Via Franco Faggiana 1668, Latina, 04100 Italy; 2 Uroresearch Association, non-profit research, Latina, Italy; 3 Department of Urology, University of Perugia, Perugia, Italy; 4 Department of Medico-Surgical Sciences and Biotechnologies, Sapienza University of Rome, Faculty of Pharmacy and Medicine, Histopathology Unit ICOT, Latina, Italy; 5 Department of Urology, Sant’Andrea Hospital, Sapienza University of Rome, Rome, Italy

**Keywords:** Non muscle invasive bladder cancer, Aspirin, Statin, Transurethral bladder resection

## Abstract

**Background:**

This study aimed to evaluate the behavior of non-muscle-invasive bladder cancer (NMIBC) in patients submitted to transurethral bladder resection (TURB) comparing subjects in chronic therapy with aspirin, statins, or both drugs to untreated ones.

**Methods:**

This retrospective study was conducted on 574 patients diagnosed with NMIBC who underwent TURB between March 2008 and April 2013. The study population was divided into two main groups: treated (aspirin and/or statins) and untreated. The treated group was further divided into three therapeutic subgroups: Group A (100 mg of aspirin, daily for at least two years); Group B (20 mg or more of statins, daily for at least two years); and Group C (100 mg of aspirin and 20 mg of statins together). The mean follow-up of patients was 45.06 months.

**Results:**

No significant differences were observed among the different groups at baseline. On multivariate analysis, statin treatment, smokers and high stage disease (T1) achieved the level of independent risk factor for the occurrence of a recurrence. When patients were stratified according to the different treatment; patients treated with statins (Group B) presented an higher rate of failure (56/91 patients; 61.5%) when compared to Group A (42/98 patients; 42.9%), Group C (56/98; 57.1%) and (133/287 patients; 46.3%). This difference corresponds to a significant difference in recurrence failure free survival (p = 0.01).

**Conclusions:**

Our results suggest that long-term treatment with aspirin in patients with NMIBC might play a role on reducing the risk of tumor recurrence. In contrast, in our investigation data from statins and combination treatment groups showed increased recurrence rates. A long-term randomized prospective study could definitively assess the possible role of this widely used drugs in NMIBC.

## Background

Urothelial bladder cancer (UBC) is common in Western countries, where it is the fourth and ninth most common cancer in men and women, respectively [1]. This frequency, coupled with the relapsing nature of UBC, means that this disease poses an enormous burden on health-care systems. Approximately 75% of newly diagnosed UBCs are non-invasive, but they have a high rate of recurrence and progression despite local treatment. The other 25% are muscle-invasive and require either radical surgery or radiotherapy, and have poor outcomes despite systemic therapy [1].

The purpose of this retrospective study was to compare the behavior of nonmuscle- invasive bladder cancer (NMIBC) between patients who had undergone transurethral bladder resection (TURB) in chronic treatment with aspirin, statins, or a combination of these two drugs and similarly operated patients who were not treated with aspirin or statins. The present study aimed to determine whether daily treatment with these medications, widely used for the prevention of cardiovascular disease, can affect the prognosis of UBC. The main target in determining the chemopreventive effect of aspirin and other non-steroidal anti-Inflammatory drugs (NSAIDs) is the inhibition of cyclooxygenase (COX) or prostaglandin-endoperoxide synthase, especially its isoform 2, which is overexpressed in many tumor cell lines, including bladder cancer (BC) [2-7]. Many studies have shown that COX inhibitors have a preventive effect and are able to induce remission of BC in animal models [7,8].

An additional aim of our study was to analyze the behavior of NMIBC in patients treated daily with statins undergoing TURB. The primary effect of statins is the reduction of low-density lipoproteins, an effect that is associated with an anti-inflammatory action. The possible role of statins in the processes of carcinogenesis and, particularly, in UBC is still not completely defined [9-12].

We also focused our attention on patients treated with both aspirin and statins. The effect of this therapeutic combination on the progression and behavior of NMIBC after TURB has not been studied previously. We compared results obtained in this population with results in the other treatment sub-populations and in untreated patients.

## Methods

This retrospective study evaluated information from 792 patients with NMIBC who underwent TURB between March 2008 and April 2013. The study was performed in accordance with the Ethical Principles for Medical Research Involving Human Subjects (World Medical Association, The Declaration of Helsinki Principles, 2000). This study was approved by the local ethics committee of Hospital [ASL Lt/no. 1674/2013], and written informed consent was obtained from all patients [or University].

The data were collected through a careful analysis of the database archive of the Department of Urology. Seventy-two patients were excluded because of lack of data regarding smoking habits, 31 were excluded because of lack of histopathological data, and 94 were not considered because of lack of personal treatment data. Among the remaining 595 patients, 21 women were excluded, for the lack of some follow-up data, in order to obtain two study groups that were statistically homogeneous and comparable.

In the remaining population of 574 patients, the following parameters were considered: age, smoking, and therapy, with particular attention to intake of aspirin and/or statins (Table 1). None of the recruited patients was previously submitted to TURB. A total of 287 patients were in treatment with aspirin (100 mg daily for at least 2 years), or with statins (≥20 mg daily for at least 2 years), or simultaneously with both drugs.

**Table 1:** **Demographic and operative data of the enrolled population**

	Total	Untreated	Treated	p - value
**Patients**	574	287	287	
**Age, yr; Mean (IQR)**	62,24 (61,2-63,2)	62,78 (61,9-63,2)	61,61 (61,2-62,6)	0,2281
**No Smokers**	158	77	81	
**Smokers**	200	133	67	0,778
**Former Smokers**	216	77	139	
**Stage**				0,513
**pTa**	284	153	131	
**Cis**	17	8	9	
**pT1**	273	126	147	
**Grade**				0,253
**Low Grade**	206	119	87	
**High Grade**	368	168	200	
**Intravesical therapy**				0.07
**BCG**	290	134	156	
**MytomicinC**	284	153	131	
**SUBGROUPS**	**Group A**	**Group B**	**Group C**	**p - value**
**Patients**	98	91	98	
**Age, yr; Mean (IQR)**	61,43 (61,2-62,8)	61,31 (61,2-62,4)	62,71 (61,9-63,3)	0,639

*IQR = Interquartile Range Group: A = Cardioaspirin; B = Statins; C = Association.*

Smoking habits of the patients were categorized as follows: current smokers (203), former smokers (189; no smoking for at least 12 months), and non-smokers (182; had smoked less than 100 cigarettes during their lifetime). The following parameters for BC were considered: number of resections, number of total lesions at the first diagnosis, and histopathological findings in recurrent tumors. All surgical specimens were staged (with re-evaluation of those resected before 2009), according to the TNM classification of the Union Internationale Contre le Cancer (UICC), updated in 2009 [13]; tumor grading was evaluated according to the 2004 World Health Organization (WHO) classification for UBC [14].

Histopathological data were evaluated according to the following criteria: the possible presence of concomitant carcinoma in situ; architecture of tumor (papillary or sessile, according to the characteristic predominant lesion); possible infiltration of lymph-vascular space (defined by the presence of tumor cells within the spaces delimited by endothelial cells); and possible tumor necrosis (the presence of coagulative necrosis in more than 10% of the lesion).

No patient received neo-adjuvant therapy, adjuvant systemic chemotherapy, or radiotherapy, but intravesical adjuvant therapy was administered according to the tumor staging and grading of each patient.

The study population was represented by two groups: treated (287 patients treated with aspirin and/or statins) and untreated (287 patients not treated with these medications). The treated group was further divided into the following subcategories: Group A, 98 patients treated with aspirin only; Group B, 91 patients treated with statins only; Group C, 98 patients treated with both aspirin and statins. The patients’ average age was 62.2 years (mean, 62.2 ± 4; range, 61.2 to 63.2).

The follow-up of the patients, as recommended by EAU guidelines [14], consisted of urinary cytology, upper urinary tract ultrasound, or computed tomography urography for the first year, and cystoscopy every three months; the follow-up evaluation was performed every six months from the second to the fifth year, then annually. During every follow-up visit, patients’ therapy was reevaluated, with particular attention to the use of aspirin and statins. The mean follow-up duration was 45.06 months (range: 16–74 months). Recurrent disease was defined according to the American Cancer Society as the return of cancer after treatment and after a period of time during which the cancer could be detected, and at the site where it began (somewhere else in the bladder or at distant sites) [14].

### Statistical analysis

Statistical analysis was performed using the S-PSS 12.0 software. Evaluation of data distribution showed a non-normal distribution of the study data set. Differences between groups of patients in medians for quantitative variables and differences in distributions for categorical variables were tested with the Kruskal Wallis one way analysis of variance and chi-square test, respectively. Using multiple logistic regression with the enter method, the statistical significant variables as assessed in the univariate analysis were entered and investigated to predict the success of the procedure. The logistic regression analysis was carried out using data from patients for whom complete data were available. Age, stage, smoking status, treatment were used as predictors of recurrence. Patients were also stratified according to the type of treatment: no treatment; aspirin (Group A); statin (Group B); aspirin + statin (Group C).

Time to failure was analyzed using Kaplan-Meier estimates. Survival times were measured in months and were censored at the date of a patient with a diagnosis of bladder cancer recurrence. An alpha value of 5% was considered as threshold for significance. Data are presented as mean ± standard deviation (SD). Odds ratios and 95% CI’s were calculated for the parameters in each group using no treatment; no smokers or low stage (Ta) as reference group.

## Results

The patient groups were similar for all considered parameters, with equal distribution of age among treated and untreated patient (p = 0.2281) and between the subgroups A, B, C (p values > 0.05). The other clinical data, treatment, and smoking habit (p = 0.788), tumor grade (p = 0.253), and stage (p = 0.513) were similar between the groups.

**Figure 1:**
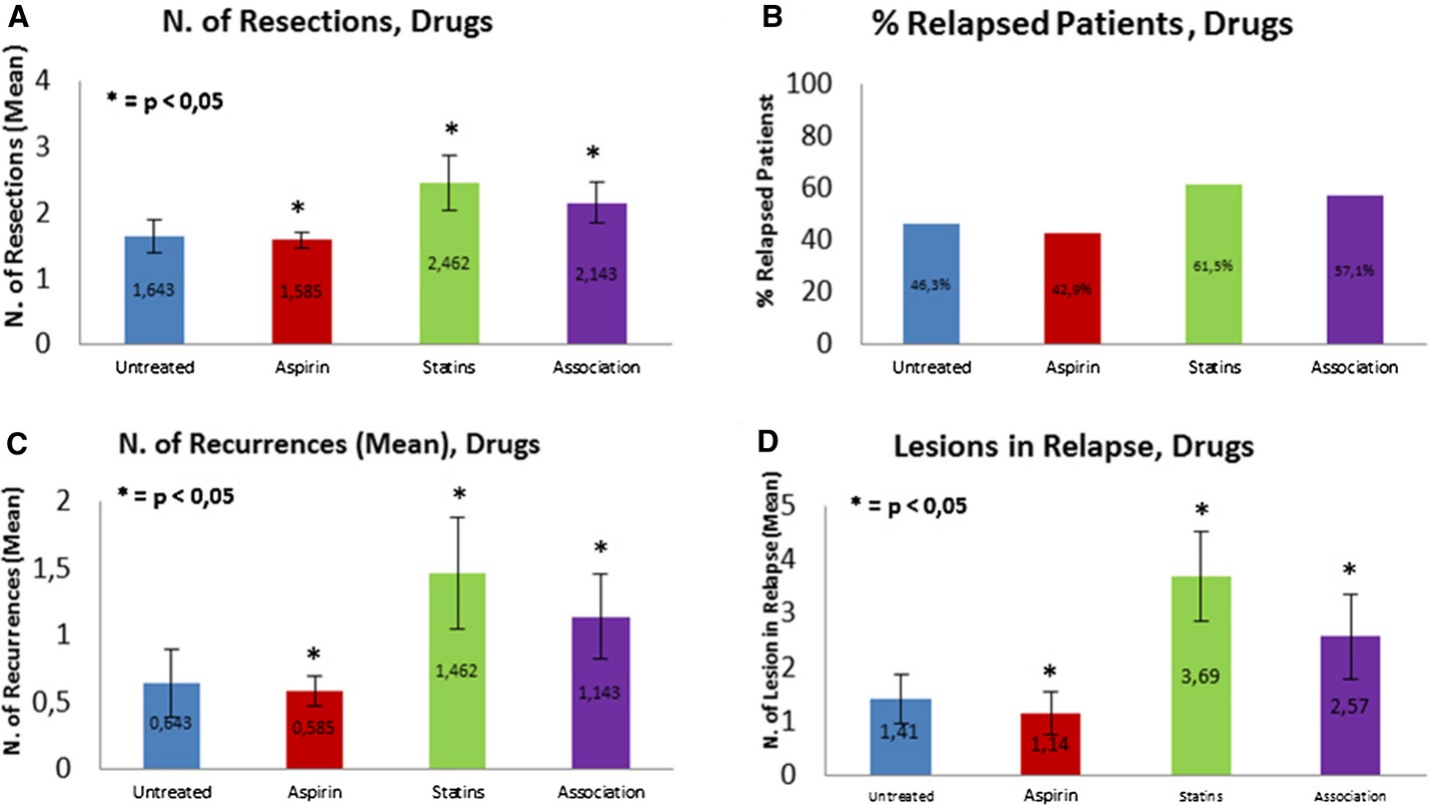
**Results from different treatment groups (untreated, aspirin, statins and association): A) number of resections; B) rate of relapsed patients; C) number of recurrences; and D) number of lesions in relapses.**

The analysis of data showed that within the treated group compared to the untreated group: Group B had the greatest number of resections (mean = 2.462 ± 0.418, p <0.0001); Group A had fewer procedures (mean = 1.585 ± 0.116) than did the entire population, particularly among the untreated group (mean = 1.643 ± 0.248). Significantly, Group C had fewer resections (mean = 2.143 ± 0.312, p < 0.05; Figure 1A) than did the statins-only group (B). The number of recurrences in the treated subgroups was as follows: Group A, 42%; Group B, 61%; and, Group C, 57% (Figure 1B).

Patients treated with statins only (Group B) had the highest number of recurrences (mean = 1.46 ± 0.418), followed by patients treated with both aspirin and statins (Group C; mean = 1.14 ± 0.312), and the untreated group (mean = 0.64 ± 0.25; p < 0.05). Group A, the aspirin group, as already evidenced by the number of resections, had fewer recurrences (mean = 0.59 ± 0.116) than did the untreated group (p = 0.042) (Figure 1C).

The correlation between the number of total lesions and the type of drug used was not statistically significant (p = 0.10). Comparing the number of lesions in recurrence relative to pharmacotherapy, the following results were obtained: subgroup A (aspirin), mean = 1.14 ± 0.397, p = 0.0242; subgroup B (statins), mean = 3.69 ± 0.827, p = 0.0188; subgroup C (both drugs), mean = 2.57 ± 0.797, p = 0.021; and untreated, mean = 1.41 ± 0.465 (Figure1D).

**Figure 2:**
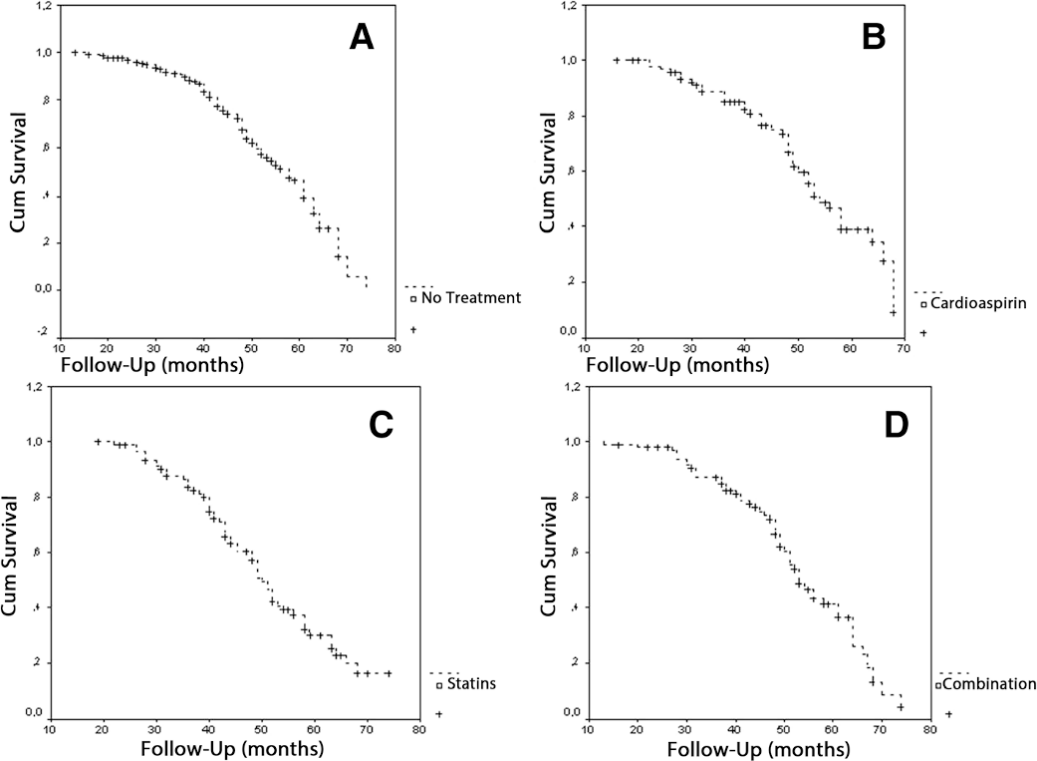
**Recurrence free survival in patients who received no treatment (A), treated with aspirin (B), with statins (C) or with aspirin and statins (D).**

On multivariate analysis (Table 2), age, statin treatment, smokers and high stage disease (T1) achieved the level of independent risk factor for the occurrence of a recurrence. When patients were stratified according to the different treatment; patients treated with statins (Group B) presented an higher rate of failure (56/91 patients; 61.5%) when compared to Group A (42/98 patients; 42.9%), Group C (56/98; 57.1%) and (133/287 patients; 46.3%). This difference corresponds to a significant difference in failure free survival (p = 0.01) as visualized in Figure 2.

**Table 2:** **Odds ratios and 95% confidence interval (CI) for predicting recurrence among patients undergoing to TURB at univariate and multivariate model**

	Univariate		Multivariate	
	OR: 95% CI	p	OR: 95% CI;	p
**Age**	0.881: 0.846-0.917	0.001	0.871: 0.833-0.911	0.001
**No Smokers**	Reference		Reference	
**Smokers**	2.17: 1.411-3.337	0.001	3.202; 1.983-5.171	0.001
**Former smokers**	2.594: 1.694-3.974	0.001	2.191; 1.382-3.478	0.001
**No Treatment**	Reference		Reference	0.261
**Aspirin**	0.868: 0.547-1.379	0.550	0.749: 0.452-1.239	
**Statins**	1.853: 1.144-3.1	0.012	1.886: 1.095-3.247	0.022
**Aspirin and Statins**	1.544: 0.972-2.452	0.66	1.394: 0.852-2.279	0.186
**Stage**	0.681: 1.208-2.332	0.002	1.915: 1.335- 2.746	0.001

## Discussion

The cause of bladder cancer is multifactorial, determined by genetic and epigenetic alterations in addition to the direct and indirect effects of multiple risk factors. The results obtained in this study demonstrate that aspirin and statins are able to modify the behavior of urothelial NMIBC.

We found a statistical significance in terms of number of bladder resections performed from 2008 to 2013 in those patients treated with aspirin and/or statins compared to those not treated with either drug group. Further evidence is given by the greater number of patients with relapse of tumor, defined as those who have required at least a second TURB, as well as the mean number of recurrences per patient, which was significantly greater in the treated patients.

The higher number of lesions in recurrence represents another important result in the treated group compared to the untreated group. In this analysis, which did not distinguish between different treatment subgroups, the prognosis of NMIBC for treated patients was worse than that for untreated patients. In a more detailed analysis, patients treated with aspirin (Group A) received significantly fewer resections than did the entire population, which is statistically significant when compared to the untreated group.

Group A (aspirin) also had fewer patients with recurrence, fewer recurrences, and fewer lesions in recurrence than did patients in the other treated groups and in the untreated group.

Similar to results reported for colon cancer [2], our study of BC has shown that regular intake of aspirin (at least 100 mg daily for a minimum of 2 years) significantly reduces the number of resections, number of patients with recurrence, total recurrences, and the number of lesions in recurrence compared to similar conditions in the entire population studied. Literature reports have documented that aspirin, as well as other NSAIDs, inhibits cyclooxygenase, especially COX-2, and is able to protect against the development of several neoplastic diseases [2,15-17].

Veitonmäki, et al., reported that the risk of prostate cancer is decreased in patients treated with acetylsalicylic acid but not with other NSAIDs [18]. In the pathogenesis of cancer, COX-2 appears to be involved in the signaling pathways linked to cell proliferation, migration, apoptosis, and tumor angiogenesis [15,19,20].

Selective inhibitors of COX-2, such as etoricoxib and celecoxib, can slow cell growth and induce apoptosis in tumor cell lines, such as BC, in both humans and animal models [8,21-23]. It has been shown that prolonged treatment with selective COX-2 inhibitors is directly related, unlike aspirin, to a significant increase in cardiovascular risk, thereby excluding the possible preventive use of these drugs [15]. The evidence of COX-2 over expression in UBC cell lines [3-7], as well as in colon carcinoma [24], is widely accepted.

Several population-based case-controlled studies have reported conflicting results about aspirin intake and BC [23-26]. The most representative study included 1514 patients in Southern California, USA, and reported a lower risk for BC in patients treated with acetylsalicylic acid [25]. These results were confirmed by two other case-controlled trials conducted in New Hampshire, USA, and Sweden [26,27]. In contrast, Daugherty, et al., conducted the largest prospective evaluation of NSAIDs and BC risk, using three well-characterized cohort studies on more than 500,000 patients; they concluded that regular use of NSAIDs, but not aspirin, reduced the risk of BC, particularly for nonsmokers [28]. Thus, we can state that there is no agreement on the role of aspirin in the biological behavior of UBC.

In our study, the daily intake of statins (20 mg or more, daily for at least 2 years) was significantly correlated with a higher number of resections (Group B) than occurred in the other treated groups, and especially than in the untreated group. In Group C (statins and aspirin), the statistical difference was less, but remained significant, in comparison to values for patients treated with statins only. Therefore, it might be assumed that the protective role of aspirin is reduced by the simultaneous intake of statins. Regarding patients with recurrence, a higher rate was observed in Group B than in the other treated subgroups and in untreated patients. This result supports the hypothesis that statins could negatively influence the protective effect of aspirin, as observed in patients treated with both medications. The number of recurrences per patient was higher in statins than in the aspirin subgroup and in the untreated subgroup. Once again, the protective effect of aspirin was strongly decreased in Group C, where the combination of drugs seems to have led to a greater number of recurrences per patient. Another significant result observed in the statin group was that of a greater number of lesions in the cases of recurrence, compared to the number in the rest of the studied population, and especially compared to that in Group A.

Statins represent a pharmacological class widely used for primary and secondary prevention of cardiovascular events. The role of statins in carcinogenesis is still controversial depending on the type of tumor. In a study conducted on 88,125 cases and 362,254 controls, long-term intake of statins (longer than 4 years) was associated with a significantly increased risk of development and recurrence of bladder and lung cancer [11,12]. In prostatic cancer, statins were associated with a reduced risk of clinical progression and mortality [29].

Few studies have analyzed the long-term effect of statins on BC. A recent meta-analysis showed that long-term statins use did not significantly affect the risk of BC, with an estimate relative risk (RR) = 1.07, and the authors highlighted a need for randomized controlled trials to determine the role of statins in BC [30]. Our results clearly showed an increased number of recurrences and lesions and an increased number of resections in patients treated with statins.

To the best of our knowledge, this is the first study that investigated the effects of combined aspirin and statin use on the behavior and progression of NMIBC. The results of Group C have demonstrated a statistically increased number of resections, rate of relapsing patients, number of recurrences, and number of lesions in relapse compared with those outcomes in the untreated group and, especially, in aspirin Group (A). Our results suggest that aspirin therapy has a protective role in BC but that statin therapy alone and statin therapy coupled with aspirin therapy do not.

The major limitations of our study are its retrospective design, the lack of stratification by type of statin, and the limited sample size. Further investigation about categories of statins in BC treatment is needed to determine the role of this drug more decisively. It is important to note that in our investigation, we analyzed a statistically homogeneous population in terms of smoking habits, which excludes that risk factor as a possible bias, as has been reported [28,30].

## Conclusion

Our results suggest that long-term treatment with aspirin in patients with NMIBC might play a role on reducing the risk of bladder tumor recurrence, average number of resections, and number of lesions in recurrence in patients who underwent TURB for NMIBC. In contrast, data from statins and combination treatment groups showed increased recurrence rates and progression of the disease. Based on this retrospective review, we planned a prospective, long-term randomized controlled study to conclusively determine the effects of these two categories of drugs on the recurrence rate and the progression of BC.

## Abbreviations

BC: Bladder Cancer
COX: Cyclooxygenase
NMIBC: Non Muscle Invasive Bladder Cncer
NSAID: Non Steroideal Anti-inflammatory Drugs
TURB: Trans Urethral Resection Bladder
UBC: Urothelial Bladder Cancer
WHO: World Health Organization

## References

[1] Burger M, Catto JW, Dalbagni G, Grossman HB, Herr H, Karakiewicz P (2013). Epidemiology and Risk Factors of Urothelial Bladder Cancer. Eur Urol.

[2] Rothwell PM, Wilson M, Elwin CE, Norrving B, Algra A, Warlow CP (2010). Long-term effect of aspirin on colorectal cancer incidence and mortality: 20-year follow-up of five randomised trials. Lancet.

[3] Mohammed SI, Knapp DW, Bostwick DG, Foster RS, Khan KN, Masferrer JL (1999). Expression of cyclooxygenase-2 (COX-2) in human invasive transitional cell carcinoma (TCC) of the urinary bladder. Cancer Res.

[4] Liebert M, Gebhardt D, Wood C, Chen IL, Ellard J, Amancio D (1999). Urothelial differentiation and bladder cancer. Adv Exp Med Biol.

[5] Shirahama T (2000). Cyclooxygenase-2 expression is up-regulated in transitional cell carcinoma and its preneoplastic lesions in the human urinary bladder. Clin Cancer Res.

[6] Shirahama T, Sakakura C (2001). Overexpression of cyclooxygenase-2 in squamous cell carcinoma of the urinary bladder. Clin Cancer Res.

[7] Hilmy M, Campbell R, Bartlett JM, McNicol AM, Underwood MA, McMillan DC (2006). The relationship between the systemic inflammatory response, tumour proliferative activity, T-lymphocytic infiltration and COX-2 expression and survival in patients with transitional cell carcinoma of the urinary bladder. Br J Cancer.

[8] Bhattacharya A, Li Y, Shi Y, Zhang Y (2013). Enhanced inhibition of urinary bladder cancer growth and muscle invasion by allyl isothiocyanate and celecoxib in combination. Carcinogenesis.

[9] Crivelli JJ, Xylinas E, Kluth LA, da Silva RD, Chrystal J, Novara G (2013). Effect of statin use on outcomes of non-muscle-invasive bladder cancer. BJU Int.

[10] da Silva RD, Xylinas E, Kluth L, Crivelli JJ, Chrystal J, Chade D (2013). Impact Of Statin Use On Oncologic Outcomes In Patients With Urothelial Carcinoma Of The Bladder Treated With Radical Cystectomy. J Urol.

[11] Vinogradova Y, Coupland C, Hippisley-Cox J (2011). Exposure to statins and risk of common cancers: a series of nested case–control studies. BMC Cancer.

[12] Friedman GD, Flick ED, Udaltsova N, Chan J, Quesenberry CP, Habel LA (2008). Screening statins for possible carcinogenic risk: up to 9 years of follow-up of 361,859 recipients. Pharmacoepidemiol Drug Saf.

[13] Sobin LH, Gospodariwicz M, Wittekind C, editors. TNM classification of malignant tumors. UICC International Union Against Cancer, ed. 7. Hoboken, NJ, USA: Wiley-Blackwell; 2009. p. 262–5.

[14] Babjuk M, Oosterlinck W, Sylvester R, Kaasinen E, Böhle A, Palou-Redorta J (2013). EAU Guidelines on Non–Muscle- Invasive Urothelial Carcinoma of the Bladder, the 2011 Update. Eur Urol.

[15] Wang D, Dubois RN (2006). Prostaglandins And Cancer. Gut.

[16] Harris RE, Beebe-Donk J, Doss H, Burr DD (2005). Aspirin, ibuprofen, and other non-steroidal anti-inflammatory drugs in cancer prevention: a critical review of non-selective COX-2 blockade (review). Oncol Rep.

[17] Jonsson F, Yin L, Lundholm C, Smedby KE, Czene K, Pawitan Y (2013). Low-dose aspirin use and cancer characteristics: a population-based cohort study. Br J Cancer.

[18] Veitonmäki T, Tammela TL, Auvinen A, Murtola TJ (2013). Use of aspirin, but not other non-steroidal anti-inflammatory drugs is associated with decreased prostate cancer risk at the population level. Eur J Cancer.

[19] Jana NR (2008). NSAIDs and apoptosis. Cell Mol Life Sci.

[20] Zha S, Yegnasubramanian V, Nelson WG, Isaacs WB, De Marzo AM (2004). Cyclooxygenases in cancer: progress and perspective. Cancer Lett.

[21] Gee J, Lee IL, Jendiroba D, Fischer SM, Grossman HB, Sabichi AL (2006). Selective cyclooxygenase-2 inhibitors inhibit growth and induce apoptosis of bladder cancer. Oncol Rep.

[22] Sabichi AL, Lippman SM (2004). COX-2 inhibitors and other nonsteroidal anti-inflammatory drugs in genitourinary cancer. Semin Oncol.

[23] Grubbs CJ, Lubet RA, Koki AT, Leahy KM, Masferrer JL, Steele VE (2000). Celecoxib inhibits N-butyl-N-(4-hydroxybutyl) - nitrosamine-induced urinary bladder cancers in male B6D2F1 mice and female Fischer-344 rats. Cancer Res.

[24] Chan AT, Ogino S, Fuchs CS (2007). Aspirin and the Risk of Colorectal Cancer in Relation to the Expression of COX-2. N Engl J Med.

[25] Castelao JE, Yuan JM, Gago-Dominguez M, Yu MC, Ross RK (2000). Nonsteroidal anti-inflammatory drugs and bladder cancer prevention. Br J Cancer.

[26] Fortuny J, Kogevinas M, Zens MS, Schned A, Andrew AS, Heaney J (2007). Analgesic and anti-inflammatory drug use and risk of bladder cancer: a population based case control study. BMC Urol.

[27] Steineck G, Wiholm BE (1995). Gerhardsson de Verdier M. Acetaminophen, some other drugs, some diseases and the risk of transitional cell carcinoma. A population-based case–control study. Acta Oncol.

[28] Daugherty SE, Pfeiffer RM, Sigurdson AJ, Hayes RB, Leitzmann M, Schatzkin A (2011). Nonsteroidal Antiinflammatory Drugs and Bladder Cancer: A Pooled Analysis. Am J Epidemiol.

[29] Brown M, Hart C, Tawadros T, Ramani V, Sangar V, Lau M (2012). The differential effects of statins on the metastatic behaviour of prostate cancer. Br J Cancer.

[30] Zhang H, Jiang D, Li X (2013). Use of Nonsteroidal Anti-Inflammatory Drugs and Bladder Cancer Risk: A Meta-Analysis of Epidemiologic Studies. PLoS One.

